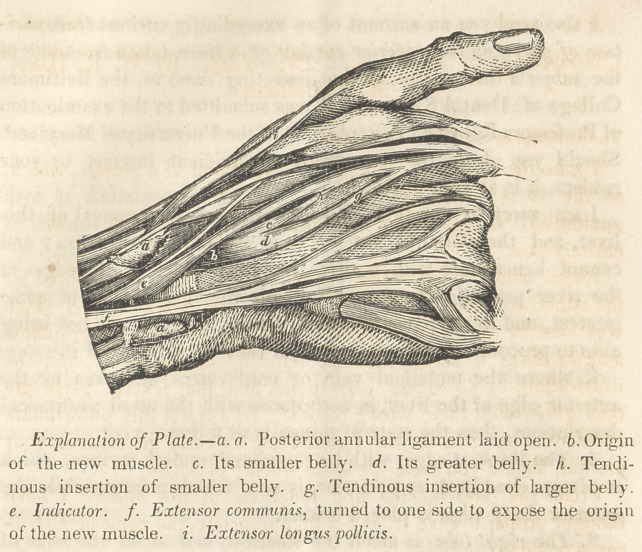# Contributions to Anatomy and Physiology

**Published:** 1850-04

**Authors:** W. R. Handy

**Affiliations:** Baltimore


### *Contributions to Anatomy and Physiology.* By W. R. Handy, M. D., Baltimore.

To the Editor of the Medical Examiner.

I take the liberty of sending you an account of a muscle, which, so far as my dissections extend, and an examination of such anatomical works, as in my opinion are entitled to credit, is entirely new.

I also send a wood-cut of the muscle, which was prepared for the American Journal and Library of Dental Science—and which was used by the same Journal, but owing to some typographical awkwardness it seems that the lettering of the plate, and the illustration, by no means correspond, and that several parts are thus given with their wrong names. To correct this unfortunate accident is one reason why I would beg an early insertion in your journal, should you deem the same worthy of notice.

From the attachments and functions of the muscle I have thought proper to give it, as not inappropriate, the name of *Extensor accessorius indicis*.

It had its *origin* on the right hand, by a delicate tendinous membrane, from the radio-carpal-articulation, behind the posterior annular ligament, and in the same groove with, and posterior to the tendons of the extensor communis and indicator, forming a fleshy bulb nearly the size of the plantaris of the leg. It soon, however, divided into two bellies—the one short and attached or *inserted* by a delicate tendon into the tendon of the indicator, near the base of the metacarpal bone of the forefinger. The other larger, and connected also to the tendon of the indicator, but near the articulation of the metacarpal bone with the first phalanx of the forefinger, and by a narrow tendon, also, as seen in the drawing.

On the left hand, the muscle had but one belly, which ended in a tendon having a similar attachment and resemblance to the larger belly upon the right.

Its *function* seems evidently to assist the indicator in the extenion of the forefinger.

*Explanation of Plate.—a. a.* Posterior annular ligament laid open. *b.* Origin of the new muscle. *c.* Its smaller belly. *d.* Its greater belly. *h.* Tendinous insertion of smaller belly. *g.* Tendinous insertion of larger belly. *e. Indicator. f. Extensor communis*, turned to one side to expose the origin of the new muscle. *i. Extensor longus pollicis.*

Remarks.—This muscle was seen upon a colored woman in the dissecting room of the Baltimore College of Dental Surgery last winter. I find that Dr. Horner, in the last edition of his Anatomy, states that the indicator muscle "is subject to many modifications;" that it is sometimes *digastric*, and sometimes *double*, in which latter case the second head goes to the middle finger. The American editor of the Dublin Dissector makes a similar remark. But a reference to the drawing clearly shows that it cannot be brought under either of these modifications of the indicator.*

* We have much pleasure in publishing the communication of Dr. Handy (which has already appeared in the Boston Med. and Surg. Journ., and the Amer. Jour. and Library of Dental Science,) for the purpose of enabling him to make the desired corrections. In one instance, however, since we have been connected with the editorial department of the Examiner, an article was sent simultaneously to two journals, (our own being one of them,) without any intimation of the fact being given to us. This we are entirely willing to believe was the result of a want of the "savoir faire" in such matters. But it is generally regarded as a want of candor on the part of the contributor, and as evincing a desire rather to publish his own name than to advance the cause of science. It is usually conceded to an editor that he shall be the

I also send you an account of an exceedingly curious *transposition of parts on the inferior surface of a liver* taken from one of the subjects this winter, in the dissecting room of the Baltimore College of Dental Surgery. It was submitted to the examination of Professors Roby and Miltenberger, of the University of Maryland. Should you deem the description of sufficient interest to your readers, it is at your disposal.

I am sorry to say that I did not witness the removal of this liver, and therefore did not see it in its natural situation; and cannot hence state with positive certainty which of the edges of the liver presented front and towards the ribs—a fact of some interest, and in reference to which, I have to regret my not being able to procure any information from those who removed it.

If where the umbilical vein or cord enters be taken as the anterior edge of the liver, in accordance with the usual anatomical descriptions, then the state of things is as follows, viz:

1. The *thickest edge*, with its round and smooth surface, which is always described as posterior, is now found in front, while the anterior sharp edge is placed behind.

2. The *right lobe* is much the smallest, and about the size of what the left usually is, while the left takes the usual size of the right.

3. The *Lobulus Spigelii* is in front, instead of behind the transverse fissure, and situated on the left, instead of the right lobe.

4. The *Inferior vena cava* also was in front of the liver, (instead of its back part,) and on the left instead of the right lobe.

If, on the other hand, the thick and round edge of the liver, together with the usual situation of the Lobulus Spigelii and ascending cava, be taken as the posterior edge, then the situation of parts is as follows, viz:

1. *Gall bladder* on the posterior edge of the left lobe, instead of anterior and inferior surface of the right.

2. *Umbilical cord* entering the centre of the posterior edge, instead of the fissure on the anterior edge.

judge as to whether an article has sufficient merit to warrant its appearance in more than one journal at the same time.

These remarks are not all intended to apply to the author of the above article, who has very candidly stated his reasons for wishing his communication to appear in our pages after their publication in others, but to those who through ignorance in such matters, might, under similar circumstances, feel offended at the non-appearance of their contributions.—Ed. Examiner.

3. *Lobulus Spigelii* passes over the transverse fissure, to the left lobe, making an arch of about an inch in breadth.

Yours respectfully,

W. R. Handy.

*Baltimore, March 7th,* 1850.

## Figures and Tables

**Figure f1:**